# Supplementary material for: The physical roles of different posterior tissues in zebrafish axis elongation
Source: Nat Commun. 2025 Feb 21;16:1839. doi: 10.1038/s41467-025-56334-7 (PMC11845790; doi:10.1038/s41467-025-56334-7)
Supplement: Supplementary file 1 — Supplementary Information [file 41467_2025_56334_MOESM1_ESM.pdf]

Supplementary Information

The physical roles of different posterior tissues in zebrafish axis elongation

Georgina A. Stooke-Vaughan[1,‡], Sangwoo Kim[1,#,‡], Shuo-Ting Yen[2,‡], Kevin Son[1], Samhita P. Banavar[3,◊], James Giammona[3], David Kimelman[4], Otger Campàs[1,2,5,6 *]

[1] Department of Mechanical Engineering, University of California, Santa Barbara, CA 93106, USA

[2] Cluster of Excellence Physics of Life, TU Dresden, 01062 Dresden, Germany

[3] Department of Physics, University of California, Santa Barbara, CA 93106, USA

[4] Department of Biochemistry, University of Washington, Seattle, WA 98195, USA

[5] Max Planck Institute of Molecular Cell Biology and Genetics, Dresden, Germany

[6] Center for Systems Biology Dresden, 01307 Dresden, Germany

[#] Present address: Institute of Mechanical Engineering, École Polytechnique Fédérale de Lausanne (EPFL), CH-1015 Lausanne, Switzerland

[◊] Present address: Department of Chemical and Biological Engineering, Princeton University, USA

[‡] These authors contributed equally

* author for correspondence: otger.campas@tu-dresden.de

Table of Contents

Supplementary Figure 1	S-2
Supplementary Notes	S-4
Cell Movement Analyzer Manual	S-5

Supplementary Figure 1:

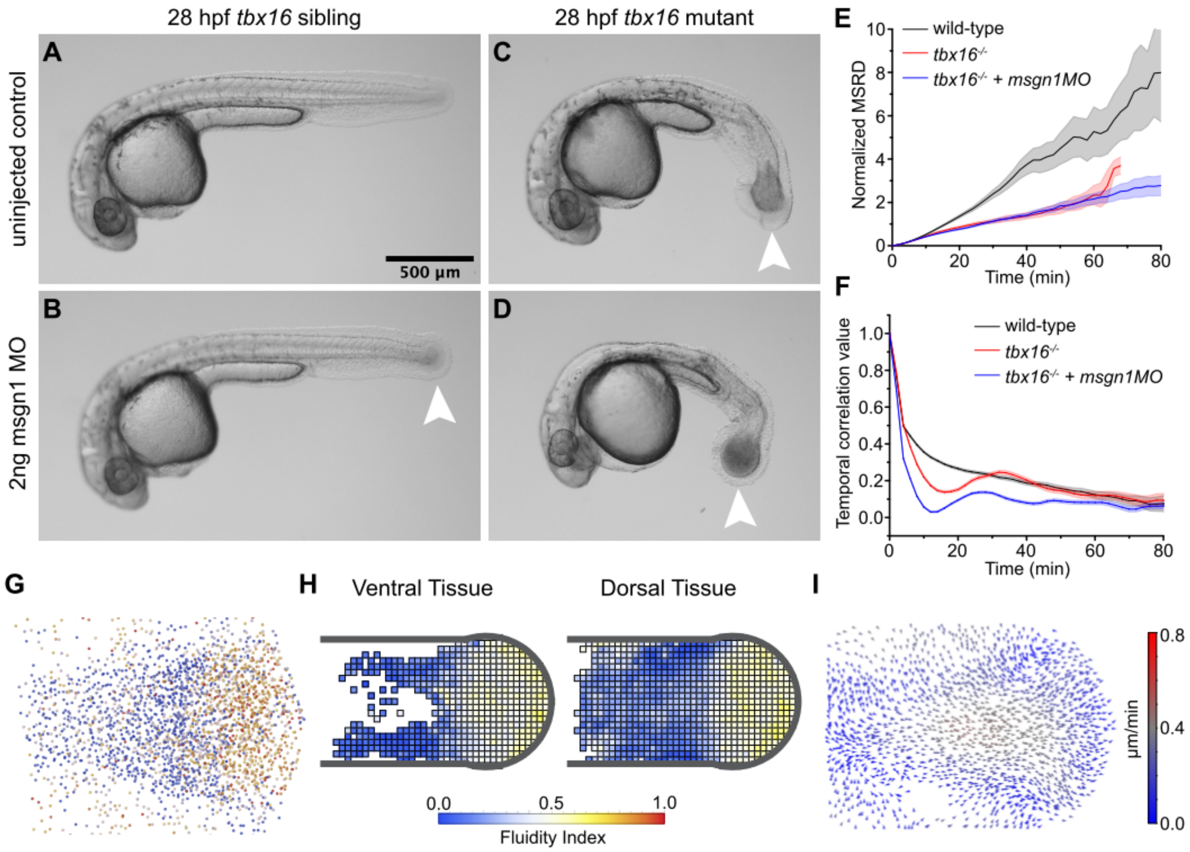

Supplementary Figure 1. Embryos without PSM show similar relative cell movements as embryos lacking PSM. A-D. Lateral views of 28hpf *tbx16* sibling and mutant embryos, with or without injection of 2ng *msgn1*MO. **B.** Sibling embryos injected with *msgn1*MO still form somites but have an increase in undifferentiated cells in the MPZ (arrowhead). **C.** *tbx16* mutant embryos fail to form trunk somites, but do form some tail somites, and have undifferentiated cells in an enlarged MPZ (arrowhead). **D.** The MPZ of *tbx16* mutant embryos injected with *msgn1*MO is larger than in *tbx16* mutants alone (arrowhead), and no somites form in the tail. **E.** MSRD in the MPZ of *tbx16* mutant injected with *msgn1*MO is not significantly different from MSRD in uninjected *tbx16* mutant, and significantly reduced from wild-type MSRD (For wild-type, n_p=3370 from N=6, for *tbx16*$^{-/-}$, n_p=1716 from N=6, for *tbx16*$^{-/-}$ + *msgn1MO*, n_p=2025 from N=3; shading: SE). **F.** Velocity temporal autocorrelation in *tbx16* mutant + msgn1MO shows a similar wave-like pattern to uninjected *tbx16* mutants (For wild-type, n_t=3831 from N=6, for *tbx16*$^{-/-}$, n_t =1925 from N=6, for *tbx16*$^{-/-}$ + *msgn1MOl*, n_t=2323 from N=3; shading: SE). **G**. Nuclear fluidity index in *tbx16* + *msgn1*MO is comparable to nuclear fluidity index in uninjected *tbx16* mutants. Nuclear FI is shown for the whole tailbud (dorsal and ventral) as there is very little ventral tissue in *tbx16* mutants injected with *msgn1*MO. **H.** Average FI (N=3 embryos) in *tbx16*$^{-/-}$ + *msgn1*MO shows a similar pattern to uninjected *tbx16*$^{-/-}$ (c.f. Fig. 2H); the ventral tissue anterior to the MPZ in *tbx16* mutants + *msgn1*MO is lateral mesoderm. **I.** Ventral velocity field for a 30µm section through a *tbx16* mutant + *msgn1*MO tailbud, showing that there is no retrograde cellular movement

from the MPZ and no region of high velocity. **G, I** show representative images from 3 *tbx16*$^{-/-}$ + *msgn1MO* embryos analyzed. Source data are provided as a Source Data file.

Supplementary Notes:

Below we provide an extensive guide on how to use the software ‘Cell Movement Analyzer’. This includes installation notes, instructions on how to use it and also descriptions of the different physical quantities measured.

Cell Movement Analyzer

Purpose

Cell Movement Analyzer (CMA) is developed to compute quantitative measures of 3D cell movements. A current version of CMA computes five measures; Mean square relative displacement (MSRD), velocity temporal autocorrelation, velocity spatial correlation (absolute), velocity spatial correlation (relative), and average normalized speed.

Installation

Python 3 and a number of python libraries such as NumPy, Pandas are required to run CMA. An easy way to install all relevant software and libraries is installing Anaconda Python 3 version from: https://www.anaconda.com/distribution/

The latest version of Anaconda is required to avoid any crashes. If Anaconda is already installed on your machine, it can be updated in Mac OS Terminal by typing "conda update --all".

CMA does not require any installation and it can be simply unzipped and used by executing run.command. CMA is developed in Mac OS environment so its compatibility on Window environment is not tested.

Input File

An input data file format in CMA follows Imaris position data file format. In the Imaris position data file, column 1,2,3,7, and 8 correspond to x position, y position, z position, time, and cell tracking ID, respectively. First 4 rows of the Imaris file are either empty rows or headers so CMA reads column 1,2,3,7, and 8 from 5th row. CMA assumes that there is no time gap for a single cell trajectory and the position data is sorted in terms of time and cell tracking ID respectively. Position data with different formats should be reformatted to conform to the Imaris position data before analysis.

All files should be organized in sub-folders under a main folder. Sub-folders categorize different sets of position files. For instance, if positions files are from three distinct regions of tissue, aPSM, pPSM, and MPZ, then three sub-folders should be created and corresponding positions files for each region should be moved to each folder accordingly. The sub-folder names are used as labels on the result plot. The main folder can contain up to 8 sub-folders.

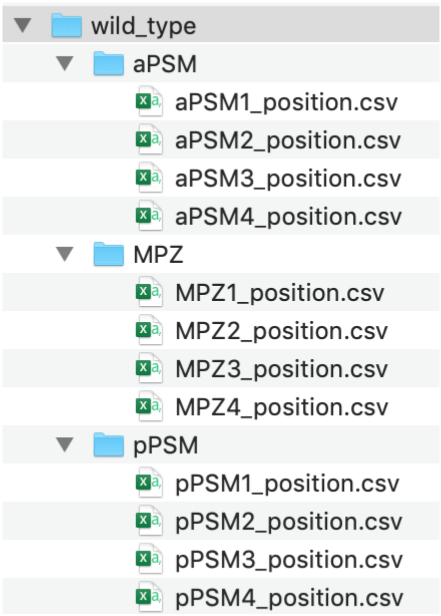

Supplementary Figure 2. Example of file and sub-folders arrangement under a main folder for analysis.

Output File

All analysis results are saved in newly created folders of given measure names under the sub-folders at the end of analysis. After the analysis is completed, one can plot the result in the GUI environment, without repeating the analysis.

Instructions

1. Double click the run.command file.
    a. May need to right click and select open if you get an error message that an application is from unrecognized developer.
2. A terminal window will open, followed by ‘Cell Tracking Calculations’ window.
3. Click ‘Import CSV’ button and then choose the main folder that contains all sub-folders of the position files
4. Check the respective boxes you want to calculate under ‘Choose what to calculate’ and then click the ‘Analyze’ button.
5. Enter the time interval in minutes.
6. Enter cell size (the average cell diameter) in µm.
    a. We used 11 µm for the wild-type 10 somite stage zebrafish tailbud data.
7. Wait until a popup message “All files have been analyzed”.
    a. The number of files analyzed for each calculation will be updated in real time.
    b. Progress or any errors will be shown in the terminal window during this time.
8. Once all files are analyzed, click any button under ‘Choose what to plot’ to plot.

9. Your plot appears at the bottom of the Cell Tracking Calculations window. You can zoom in on regions, set the start and end points of the axes, export the plot to a graphic file, etc. using the toolbar along the top of the plot.
   a. Plot buttons are from left to right:
      i. Reset plot to 'home' state
      ii. Undo
      iii. Redo
      iv. Move plot by dragging
      v. Zoom into box
      vi. Change borders and spacing around plot
      vii. Figure Options
         1. Change title,
         2. Axes start and end points
         3. Axes labels
         4. Axes scale (linear vs log)
      viii. Save/Export plot
10. The results will also output as csv files to the corresponding measures chosen within each folder analyzed.

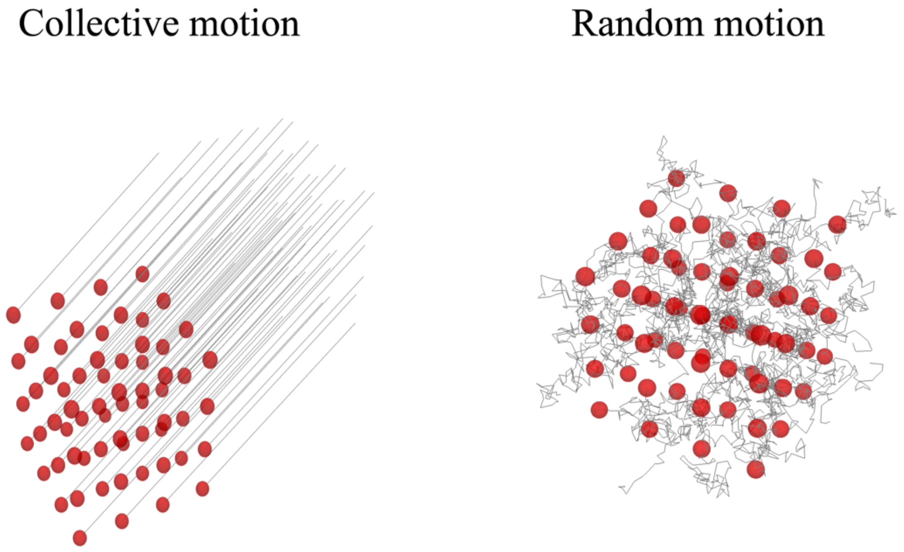

Supplementary Figure 3. Cell movement examples, one with collective motion only and the other with random motion only. Red spheres and gray lines are initial positions and trajectories of individual cells respectively.

Measurement Description

1. Mean squared relative displacement (MSRD)

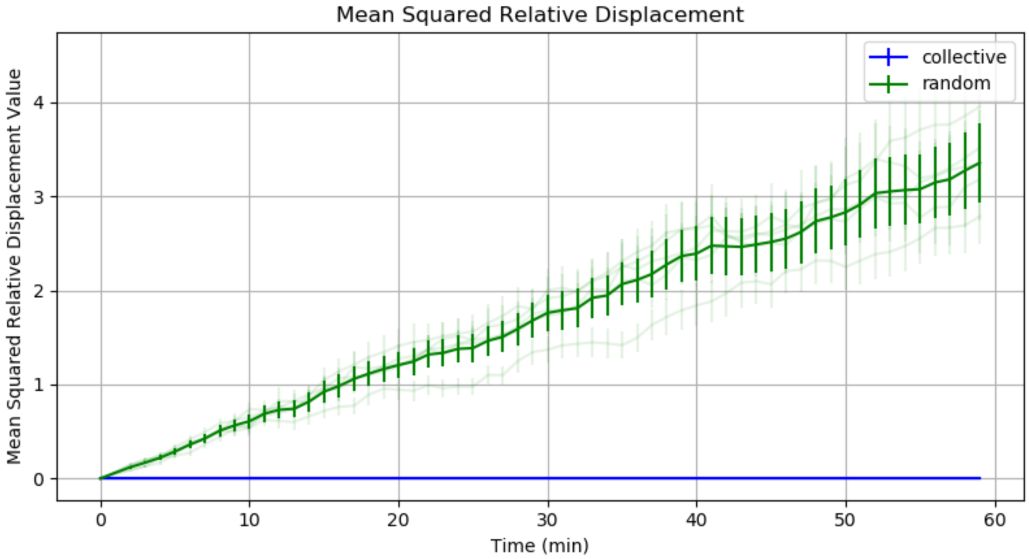

Supplementary Figure 4. MSRD results of the two example samples in Supplementary Figure 3.

MSRD quantifies relative deviation of cell positions over time with respect to the neighboring cell position and it is closely related to cell rearrangement. In general, *MSRD*>1 hints at structural rearrangement of many cells for a given time scale that is signature of fluid-like behavior of biological tissues.

Mean squared displacement (MSD) is widely used to quantify deviation of cell positions with respect to a reference position, which is an initial position of cell trajectory in general. While MSD is a powerful measure to quantify degree of cell displacement over time, it is not an appropriate measure to identify structural rearrangements for systems under collective motion. For systems that exhibit a higher degree of collective motion, MSD can increase significantly over time while local neighbor exchange is negligible. Hence, an alternative measure is essential to properly detect structural rearrangement of cellular systems.

Mean squared relative displacement (MSRD) tracks a distance vector between a pair of neighboring cells over time so local cell rearrangements can be properly quantified even for the systems under collective motion. Suppose that cell *i* appears in the system at time t_0, the nearest neighbor, cell *j*, can be identified based on the Euclidean distance at t_0. The distance vector between cell *i* and cell *j* at t_0 is defined as below.

$$\mathbf{r}_{ij}(t_0) = \mathbf{r}_j(t_0) - \mathbf{r}_i(t_0)$$

Along the duration of trajectories for both cell *i* and cell *j*, the change of the distance vector can be computed for each time lag *t.* MSRD is computed by averaging the square of the change of the distance vector over all cells for a given time lag, *t* and it is normalized by the square of cell size, L^2.

$$\mathrm{MSRD}(t) = \frac{< \left|\mathbf{r}_{ij}(t+t_0) - \mathbf{r}_{ij}(t_0)\right|^2 >_{\text{all neighbor pairs}}}{L^2}$$

2. Velocity temporal autocorrelation (VTA)

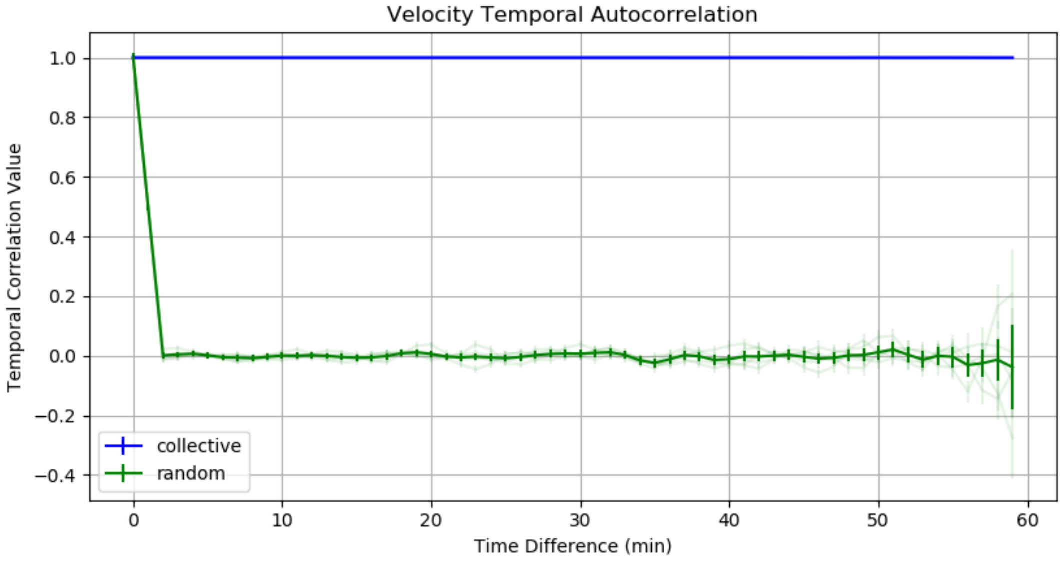

Supplementary Figure 5. Velocity temporal autocorrelation results of the two example samples in Supplementary Figure 3.

Velocity temporal autocorrelation (VTA) is a measure of the persistence of individual cell movements, that is if a cell continues moving in the same direction over time. A cell that continues moving with the same velocity over the course of the timelapse will have VTA of 1, whereas a cell that displays random changes in speed and direction will have VTA of 0. The average VTA of all tracked cells is plotted, with averaged correlation over cells on the y-axis and time on the x-axis. VTA at time 0 is always 1 due to normalization, and generally decays exponentially over time. The rate of decay of VTA allows the timescale over which a cell changes the direction of movement to be inferred and allows comparison of persistence of cell motion between samples/tissues.

VTA is the correlation of velocity values of a single cell with a time lag. From a given cell trajectory, velocity values can be computed using central difference formula. The velocity temporal autocorrelation with the time lag, τ, can be computed by averaging dot products of two velocity values over all initial time and all cells and it is normalized by the correlation value with $\tau = 0$.

$$C_T(\tau) = \frac{\langle \mathbf{v}_i(t+\tau) \cdot \mathbf{v}_i(t) \rangle_{t,i}}{\langle \mathbf{v}_i(t) \cdot \mathbf{v}_i(t) \rangle_{t,i}}$$

3. Velocity spatial correlation (Absolute)

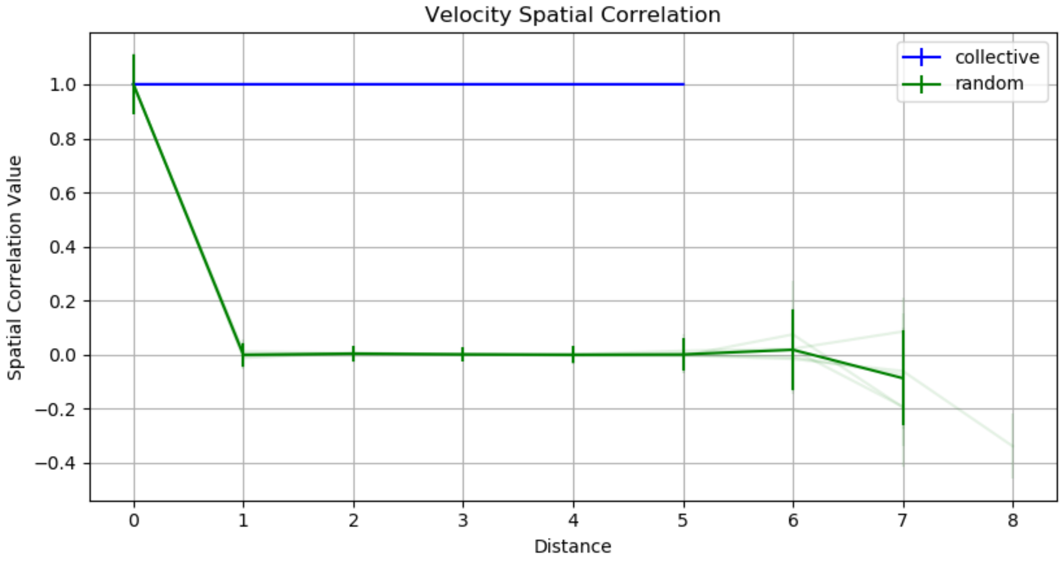

Supplementary Figure 6. Velocity spatial correlation results of the two example samples in Supplementary Figure 3.

Velocity spatial correlation (VSC) is a measure of how much a cell is moving with the same velocity as its neighboring cells. If all the cells in a tissue are moving in the same direction with the same velocity, then VSC will be 1, whereas if all the cells are moving randomly with respect to their neighbors, VSC will be 0.

VSC is plotted with the averaged correlation value over time and cells on the y-axis, and distance, normalized by the cell size input by the user, on the x-axis. At distance 0 correlation is always 1, but then decays exponentially as distance is increased. The rate of this decay can be used to infer the size of collectively moving cell groups, and to compare different samples to see if there is a change in the distance over which cell movement is correlated.

VSC is the correlation of velocity values of a pair of cells with a distance, *r*. VSC can be computed by averaging dot products of velocity values of two cells at a given time with a distance, *r*. The distance value is generally a continuous variable so the averaging for the distance *r* contains all pairs of cells with distance between $r - \Delta r$ and $r - \Delta r$ where Δr is chosen to be a half of cell size. The correlation value is normalized by the correlation value with *r=0*.

$$C_S(r) = \frac{\langle \mathbf{v}_i(t) \cdot \mathbf{v}_j(t) \rangle_{t, r-\Delta r < |\mathbf{r}_{ij}(t)| < r+\Delta r}}{\langle \mathbf{v}_i(t) \cdot \mathbf{v}_j(t) \rangle_{t, 0 < |\mathbf{r}_{ij}(t)| < \Delta r}}$$

The correlation value is equal to one at *r=0* due to the normalization and it generally decays to zero over distance. The group size of collective motion can be identified from the rate of the correlation value decay.

4. Velocity spatial correlation (Relative)

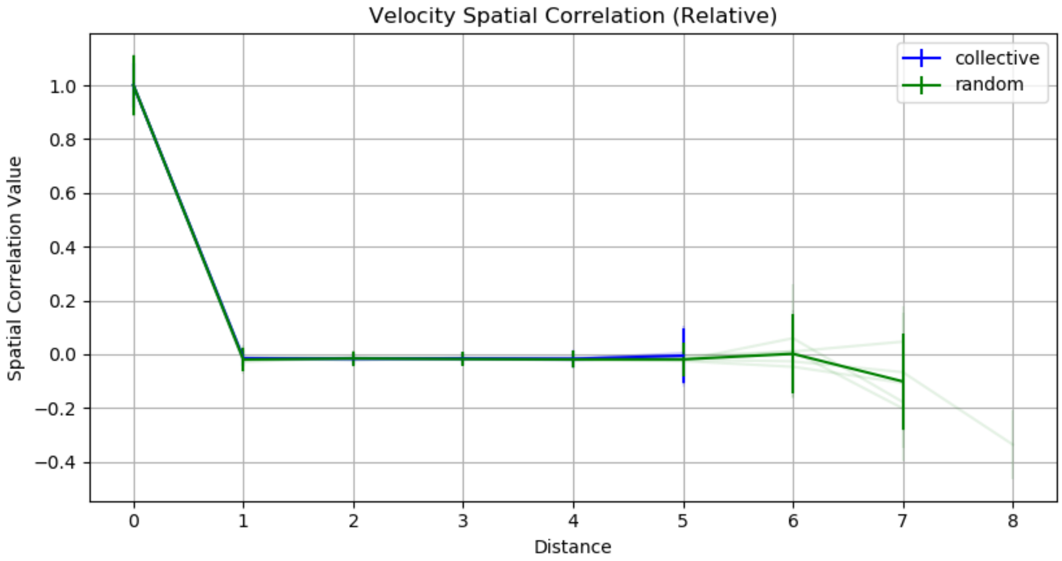

Supplementary Figure 7. Velocity spatial correlation(relative) results of the two example samples in Supplementary Figure 3.

Velocity spatial correlation relative (VSCR) is the same as VSC, but the average velocity of the system as a whole is subtracted from all cells before VSCR is calculated. In a system with collective cell motion VSC does not decay to 0, but to a value that reflects the spatial correlation caused by collective motion in the system. By calculating VSCR the same system will have a correlation that decays to 0, allowing the rate of decay to be used to infer and compare the length scales of regions that show correlated cell movement within the collective motion of the system as a whole.

VSCR is the correlation of velocity values subtracted by mean velocity value and its calculation is analogous to VSC except VSCR utilizes relative velocity to the mean velocity.

$$C_{S,r}(r) = \frac{\langle (\mathbf{v}_i(t) - \langle \mathbf{v}_i(t) \rangle_i) \cdot (\mathbf{v}_j(t) - \langle \mathbf{v}_j(t) \rangle_j) \rangle_{t, r-\Delta r < |\mathbf{r}_{ij}(t)| < r+\Delta r}}{\langle (\mathbf{v}_i(t) - \langle \mathbf{v}_i(t) \rangle_i) \cdot (\mathbf{v}_j(t) - \langle \mathbf{v}_j(t) \rangle_j) \rangle_{t, -\Delta r < |\mathbf{r}_{ij}(t)| < \Delta r}}$$

5. Average Normalized Velocity

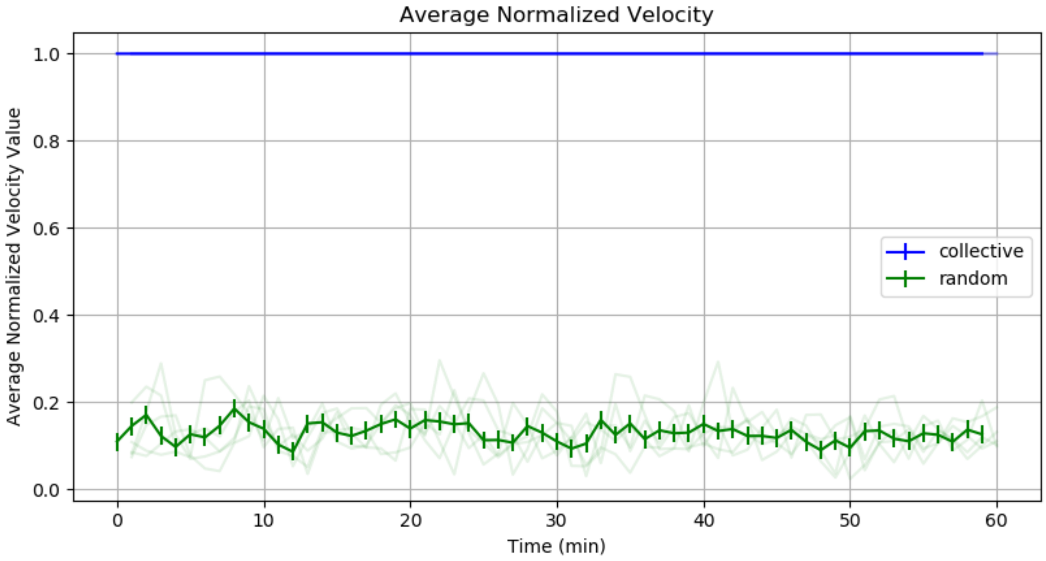

Supplementary Figure 8. Average normalized velocity results of the two example samples in Supplementary Figure 3.

Average Normalized Velocity (ANV) is a measure of the level of collective cell motion in a tissue as a whole. If ANV is 0 then this implies cell movement in the tissue is entirely random and there is no collective cell motion, whereas a tissue where all cells are moving in the same direction will have an ANV of 1. However, because ANV averages across all the cell movements in the region of interest, small sub-regions of collective motion, or sub-regions where there are opposing collective cell motions within the region of interest will be missed, and not increase the value of ANV. For ANV to be most informative, it is important to use a region of tissue where all cells are moving in a similar manner. ANV is also system-size dependent so non-zero value can be obtained from a system with random motion if the system contains a small number of cells.

ANV is the magnitude of mean velocity for a given time normalized by mean speed. Mean velocity can be computed by vector sum of individual cell velocity divided by number of cells and mean speed can be computed by sum of individual cell speed divided by number of cells.

$$v(t) = \frac{|\langle \mathbf{v}_i(t) \rangle_i|}{\langle |\mathbf{v}_i(t)| \rangle_i}$$
