# Supplementary material for: The physical roles of different posterior tissues in zebrafish axis elongation
Source: Nat Commun. 2025 Feb 21;16:1839. doi: 10.1038/s41467-025-56334-7 (PMC11845790; doi:10.1038/s41467-025-56334-7)
Supplement: Supplementary file 2 — Reporting Summary [file 41467_2025_56334_MOESM2_ESM.pdf]

nature research

Corresponding author(s): Otger Campas

Last updated by author(s): Jan 7, 2025

Reporting Summary

Nature Research wishes to improve the reproducibility of the work that we publish. This form provides structure for consistency and transparency in reporting. For further information on Nature Research policies, see our Editorial Policies and the Editorial Policy Checklist.

Statistics

For all statistical analyses, confirm that the following items are present in the figure legend, table legend, main text, or Methods section.

n/a	Confirmed	
☐	☒	The exact sample size (*n*) for each experimental group/condition, given as a discrete number and unit of measurement
☐	☒	A statement on whether measurements were taken from distinct samples or whether the same sample was measured repeatedly
☐	☒	The statistical test(s) used AND whether they are one- or two-sided<br>*Only common tests should be described solely by name; describe more complex techniques in the Methods section.*
☒	☐	A description of all covariates tested
☒	☐	A description of any assumptions or corrections, such as tests of normality and adjustment for multiple comparisons
☐	☒	A full description of the statistical parameters including central tendency (e.g. means) or other basic estimates (e.g. regression coefficient) AND variation (e.g. standard deviation) or associated estimates of uncertainty (e.g. confidence intervals)
☐	☒	For null hypothesis testing, the test statistic (e.g. *F*, *t*, *r*) with confidence intervals, effect sizes, degrees of freedom and *P* value noted<br>*Give P values as exact values whenever suitable.*
☒	☐	For Bayesian analysis, information on the choice of priors and Markov chain Monte Carlo settings
☒	☐	For hierarchical and complex designs, identification of the appropriate level for tests and full reporting of outcomes
☒	☐	Estimates of effect sizes (e.g. Cohen's *d*, Pearson's *r*), indicating how they were calculated

Our web collection on statistics for biologists contains articles on many of the points above.

Software and code

Policy information about availability of computer code

Data collection	A laser scanning confocal microscope (Zeiss, LSM710; Zen Black) was used for all in vivo imaging experiments. A laser scanning confocal microscope (Zeiss, LSM980; Zen Black) was used for 3D scans of fixed tailbud tissue. A custom-written software (LabVIEW, National Instruments) was used to synchronize the movement of the magnet array with image acquisition (using the confocal microscope), and to control both the translation and rotation stages during magnetic actuation experiments.
Data analysis	Commercial software used to analyze data: Imaris (9.3 Bitplane), Matlab 9.11 and 9.14 and Mathematica 12 (MathWorks), Python 3.8, Origin 2017. ImageJ and the Tissue Analyzer Fiji plug in was also used to analyze data. The aspect ratio of the droplet ellipsoidal deformation was determined using a previously-developed software in LabVIEW (National Instruments), publicly available from ref. 56.

For manuscripts utilizing custom algorithms or software that are central to the research but not yet described in published literature, software must be made available to editors and reviewers. We strongly encourage code deposition in a community repository (e.g. GitHub). See the Nature Research guidelines for submitting code & software for further information.

Data

Policy information about availability of data

All manuscripts must include a data availability statement. This statement should provide the following information, where applicable:

- Accession codes, unique identifiers, or web links for publicly available datasets
- A list of figures that have associated raw data
- A description of any restrictions on data availability

Source data are provided with this paper.

Field-specific reporting

Please select the one below that is the best fit for your research. If you are not sure, read the appropriate sections before making your selection.

☒ Life sciences ☐ Behavioural & social sciences ☐ Ecological, evolutionary & environmental sciences

For a reference copy of the document with all sections, see nature.com/documents/nr-reporting-summary-flat.pdf

Life sciences study design

All studies must disclose on these points even when the disclosure is negative.

Sample size	No statistical methods were used to predetermine sample sizes, but our sample sizes are similar to those reported in previous publications (refs. 10, 11, 16).
Data exclusions	In ferrofluid droplet experiments, we excluded data from droplets that did not deform above the yield stress of the tissue. Image stacks for Cdh2 level quantification were excluded when the mounted embryo was tilt or not centered. Otherwise, no samples were excluded.
Replication	Independent experiments were performed and statistical analysis done independently of these data sets. The results obtained were the same within error bars in different, independent data sets.
Randomization	No specific experimental groups were defined.
Blinding	Analysis was done by automated software which was blind to data collection, with the exceptions of manual tailbud segmentation and determination of body axis elongation speed.

Reporting for specific materials, systems and methods

We require information from authors about some types of materials, experimental systems and methods used in many studies. Here, indicate whether each material, system or method listed is relevant to your study. If you are not sure if a list item applies to your research, read the appropriate section before selecting a response.

Materials & experimental systems

n/a	Involved in the study	
☒	☐	Antibodies
☒	☐	Eukaryotic cell lines
☒	☐	Palaeontology and archaeology
☐	☒	Animals and other organisms
☒	☐	Human research participants
☒	☐	Clinical data
☒	☐	Dual use research of concern

Methods

n/a	Involved in the study	
☒	☐	ChIP-seq
☒	☐	Flow cytometry
☒	☐	MRI-based neuroimaging

Animals and other organisms

Policy information about studies involving animals; ARRIVE guidelines recommended for reporting animal research

Laboratory animals	Zebrafish (Danio rerio) were used in this study. Since only embryos were studied, sex-specific experiments were not necessary, as zebrafish embryos at the studies stage have not yet undergone sex determination.
Wild animals	None.
Field-collected samples	None.
Ethics oversight	Experiments were performed following all ethical regulations and according to protocols according to protocols approved by the IACUC of UC Santa Barbara and the European Union (EU) directive 2010/63/EU as well as the German Animal Welfare act.

Note that full information on the approval of the study protocol must also be provided in the manuscript.
